# Review on contribution of indigenous food preparation and preservation techniques to attainment of food security in Ethiopian

**DOI:** 10.1002/fsn3.1274

**Published:** 2019-11-27

**Authors:** Chala Gowe Kuyu, Tizazu Yirga Bereka

**Affiliations:** ^1^ Department of Postharvest Management Jimma University, College of Agriculture and Veterinary Medicine Jimma Ethiopia

**Keywords:** Ethiopia, food processing and preservation, food security attainment, storage

## Abstract

One of the main challenges facing Ethiopia today is ensuring food security, as the country demands more food than before with the increase in population. Although the country's production is much lower than the national demand, there are high postharvest food losses, largely due to limited food processing, preservation, and storage capacity. Universities and research centers in the country had been done and doing research on the postharvest activities to assure effective and sustainable methods of food security enhancement strategy. In contrast, most of the strategies and technologies they develop never get implemented by farmers as they still rely on indigenous knowledge for postharvest activities. Although people are using indigenous knowledge, their contributions and potentials in food processing, preservation, and storage are underestimated. However, indigenous methods of food preparation, preservation, and storage are time tested and have been used by locals people over generation to preserve their produce after harvest, thereby serving as a survival strategy. Moreover, the country is blessed with various types of wild edible plants and also possesses diverse indigenous knowledge systems for their processing, preservation, and storage. These indigenous foods are inexpensive to use, safe, nutritious, and thus boosting overall food security. Therefore, the aim of this review paper is to document indigenous knowledge of food storage, processing, and preservation in the country. This could help as a gateway to verify and support indigenous knowledge with latest technologies and promote their role in attainment of food security.

## Graphical abstract

Although different scholars are conducting research toward postharvest reduction to assure effective and sustainable methods of food security, people still relay on indigenous practices for postharvest activities. However, the role of indigenous knowledge in food processing, preservation, and storage is ignored in the country. Therefore, documentation and upgrading this knowledge for food security attainment deserve special attention as they encompass knowledge that may be especially valuable in times of crisis or adaptation to changing conditions.

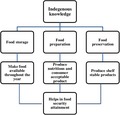

## 1 INTRODUCTION

Ethiopia is the second‐most populous country in Africa next to Nigeria with a fast population growth rate but with huge food deficit gap (Gebreselassie, 2006; Mohammed, Woldeyohannes, Feleke, & Megabiaw, 2014). The country is the first as the worst of all African countries as nearly 33 million people are suffering from chronic undernourishment and food insecurity (Food & Agriculture Organization, 2014). Among people suffering from chronic undernourishment, the largest group is rural people with insufficient assets to produce and purchase food (Endalew, Alemu, & Bizuayehu, 2015). About 33.6% of the Ethiopian population are living below the poverty line and cannot meet their daily minimum nutritional requirement of 2,200 calories (Godfray et al., 2010). According to the 2014 Central Statistical Agency report, nationally 40%, 25%, and 9% of children under age five were stunted, underweight, and wasted, respectively (Central Statistical Agency, 2014). Protein–energy malnutrition and the various micronutrient deficiency disorders including vitamin A deficiency, nutritional anemias due to iron deficiencies, folic acid and vitamin B12, and iodine deficiency disorders remain important public health problems (Godfray et al., 2010).

Although the country is facing food insecurity problem, there are high postharvest food losses, largely due to limited food processing and preservation capacity. Different scholars have been estimated that the country's average postharvest losses reach up to 15% for cereal crops, 30%–80% for fruit and vegetables and 40% for milk and its derivatives (Derege & Getachew, 1989; Getachew, 2003; Kuyu & Tola, 2018).

It is obvious that increasing production is one aspect of fulfilling food demand; on the other hand, failure to reduce postharvest loss can also decline food availability not only due to high commodity loss but also weakening the purchasing power of the family income from the diminished market opportunities. Reducing postharvest losses instead of increasing production is not only making more food available to consumers, it also saves limited resources and reduces environmental pollution due to intensive farming (Zorya et al., 2011). Therefore, reducing postharvest losses through food preservation and processing seems a much better option to make more foods available without increasing the pressure on the limited natural resources (Hodges, Buzby, & Bennett, 2011).

One way of reducing this huge postharvest loss and improve food security is the recognition, promotion, and utilization of indigenous knowledge, skills, and practices in food handling, processing, preservation, and storage (Asogwa, Okoye, & Oni, 2017). Indigenous knowledge is the knowledge indigenous people are known and practices over generations and proved flexible enough to cope with the change through trial and error (Melchias, 2001). World Bank has recognized that indigenous knowledge is innovative and unique among the local producers and can help in fighting against hunger and malnutrition (World Bank, 1998). This is because indigenous knowledge has been used by communities as the basis for decisions pertaining to food security at the local level.

**Figure 1:**
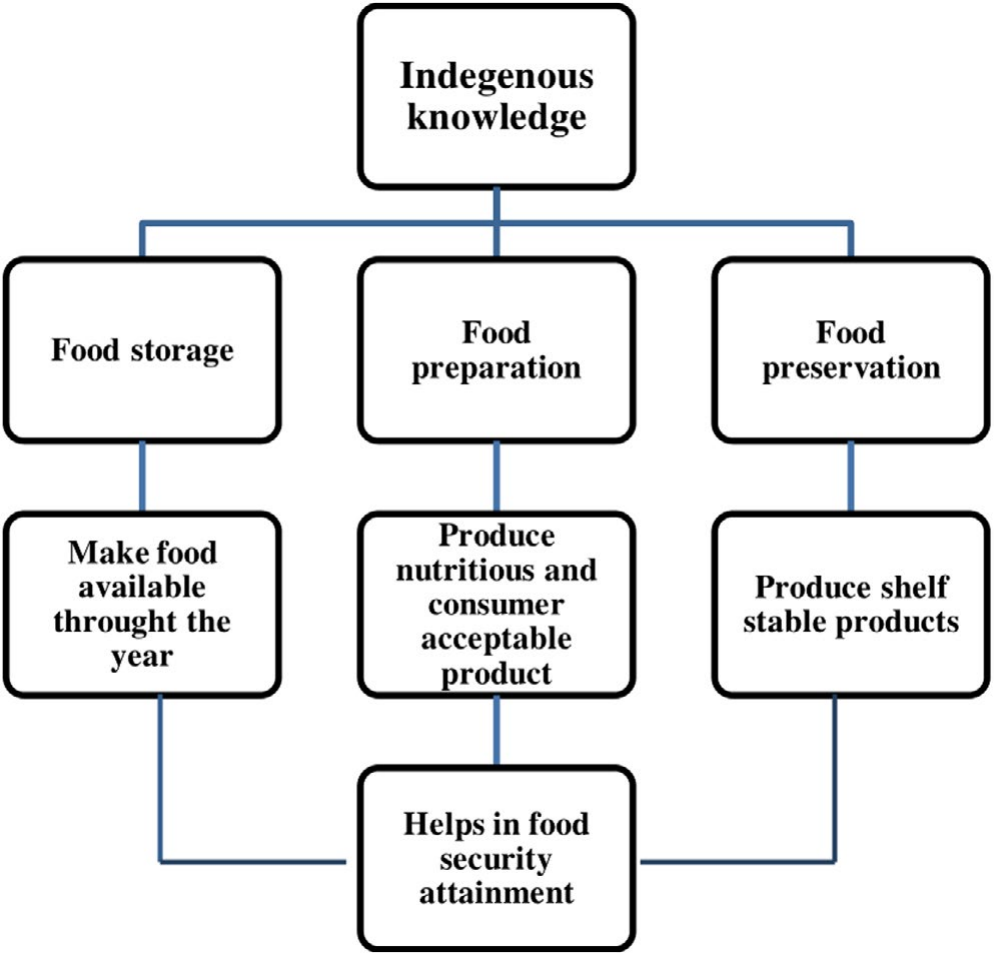
The conceptual framework of indigenous practices contribution in the attainment of food security

The Ethiopian traditional food handling, preparation, and preservation practices are the indigenous knowledge that are handed over to the present generation, which are invaluable and intangible assets as they are the outcome of repeated research and practical experiment by many generations (Ethiopian herald,). Much of the indigenous knowledge's nearby the communities are easily accessible to achieve medium‐ and long‐term food preservation and a more variable diet and also possess important cultural identification values. Thus, building on indigenous knowledge systems will empower local communities in Ethiopia, enabling them to shape their own food security agenda by actively participating in it. Although different scholars are conducting research toward postharvest loss reduction to ensure effective and sustainable methods of food security is available in the country, people are still relay on indigenous practices for food storage, food preparation, and preservation activities. The conceptual framework of indigenous practices contribution in the attainment of food security is indicated in Figure 1.

However, the role of indigenous knowledge in food processing, preservation, and storage is ignored in the country and its documentation and dissemination remain a big challenge. Therefore, documentation and dissemination of this knowledge and practices deserve special attention as they encompass knowledge, which may be especially valuable in times of crisis or adaptation to the changing conditions. This could help researchers and development mediators as a gateway to verify, support indigenous knowledge with the latest technology to cope up with the harshness of nature and promote their role in the attainment of food security. In light of the above information, this paper reviews the available literatures on the role of indigenous knowledge in the attainment of food security.

## 2 INDIGENOUS KNOWLEDGE ON POSTHARVEST STORAGE, PRESERVATION AND VALUE ADDITION

### 2.1 Indigenous practices on grains storage

In Ethiopia, the greater proportion of grains is produced by resource‐poor farmers in remote villages and they store grains to facilitate the uniform supply of food throughout the year, to save it for unforeseen events and to sell on higher prices. During storage, farmers are using a poor postharvest storage facility, which often makes them incur high postharvest losses (Sori, 2014). Among biotic factors, storage insects are the primary causes of loss for grains in storage and constitute a great constraint to the realization of food security. In spite of the use of all available means of plant protection, the overall annual average damage caused by insect pests is estimated to be 10%–40% worldwide (FAO [Food and Agriculture Organization of the United Nations], 1997; Satin, 1997) and 5%–30% in Ethiopia (Abraham, 1991; Befikadu, 2014; Emana, 1999). To overcome the above‐stated losses, smallholder farmers in Ethiopian use a number of indigenous practices to ensure a long shelf life of grains after harvesting and attain food security. Some of the indigenous storage practices used by smallholder households are described and discussed below.

#### 2.1.1 The use of cultural and physical methods of pest control

Farmers recognized that fields close to storage sites were more infested than others and most farmers’ isolate host crop fields from storage sites to reduce the level of initial infestations (Tadesse & Eticha, 2003). Proper drying is also another cultural control method in which farmers in Ethiopia used to control pest infestation by reducing moisture content and temperature that increase the effectiveness of insecticide protectants (Emana, 1993). Cleaning the storage structure, sealing of cracks, crevices, and holes present in the floors and proper hygiene and sanitation to prevent pest damage in the warehouses are other cultural practices used by smallholder farmers. The sacks of jute, sisal, and nylon should be washed and boiled in hot water to kill off pests or their eggs and larvae and then dried prior to use (Danilo, 2003). High temperature due to boiling or direct solar radiation may kill the developing larvae in the seed. Black polyethylene bags enveloped with another transparent polyethylene sheets killed a higher proportion (90.5%) of weevils as compared to the check (sisal sack = 30%) after 24‐hr exposure to the sun (Fentahun, 1995). All eggs and adult weevils were killed when infested grain was heated at 60°C for 2 hr and at 70°C and 80°C for 1 hr (at the initial moisture contents of 13% and 16%, respectively); larvae were killed only at 70 and 80°C (Mohammed, 1996). High‐density black polyethylene sheet has collected the highest solar heat (63°C) and caused 100% mortality in the maize weevil after 3½ hr. of exposure to the sun (Adane, Seneshaw, & Fentahun, 1997). However, temperature, time, and depth of grain layering for effective disinfestations are to be determined before recommendation for practical use.

Most farmers store their maize with cobs as with complete husk covers protected from insect damage (Abraham, 1991, 1997). Some farmers tie husks at the tip of cobs together in such a way that the cob is covered completely. Farmers suspend a bunch of cobs in smoke over fire, and such stored maize was less experienced damage (Abraham, 1991; Emana, 1993). This retards development and prevents re‐infestation by migrating insects because of the heat and smoke accelerate drying and reduce moisture content of the cobs to 8%–10%. Farmers also used common ingredients such as table salt that has hygroscopic and insecticidal properties. It absorbs moisture and helps the grain to keep dry and aid in its safe storage by retarding spoilage (Gupta, Verma, Bareth, & Mathur, 2010).

In order to control the storage pests particularly weevil, farmers in Ethiopia use different storage treatments. Among others are smoking the store with hot pepper, rubbing the inner wall and base of the store with flour of hot pepper. Moreover, farmers in central and northern Ethiopia use dry straw of Teff or other cereals at the base of the pit in order to control the direct contact of the grain with the base soil to reduce mold formation. Free movement of the adults for oviposition is prevented by the substance such as filling ash in the intergranular spaces. A study was conducted to compare wood ash, sand, tobacco dust, sawdust, neem seed powder, and pirimiphos‐methyl in the laboratory at Awassa (southern Ethiopia) and reported that tobacco dust was superior to all treatments in terms of damage caused by the Angoumois grain moth larvae and seed germination, followed by their mixtures. However, treatment with tobacco dust left undesirable taste (Emana, 1993; Getu & Amlak, 1995).

#### 2.1.2 The use of botanical plants in the storage for postharvest pest management

About one thousand species of insects have been found associated with stored products. Among many stored insects the most well known are weevils, bruchids, larger grain borer, and grain moth (Tefera, Mugo, & Likhayo, 2011). Weevils (*Sitophilus Species*) are one of the major and predominant pests among storage pests of grains and contribute to food insecurity and low farm incomes in Ethiopia (Keba & Sori, 2013).

Although satisfactory pest control has been obtained by the use of synthetic pesticides, their adverse effects on environment and development of resistant pest strains and residues in food crops are the major drawback of synthetic pesticides. As an alternative to address the issue of grains loss to insect pest damage in storage, subsistent farmers in Ethiopia treat grains with botanical plants available in their locality. Botanicals are plants or plant‐derived products, which have active ingredients for the control of storage pests (Said & Pashte, 2015). Because of their bioactive components, botanical plants have a property to kill and repel pests; affect insect growth and development, antifeedant, and arrestant effects; and have antifungal, antiviral, and antibacterial properties against pathogens (Prakash & Rao, 1997).

Some of specific botanical plants, which have been documented as grain protectant in the storage, include the following: basil powder as insecticide against maize weevil (Mwangangi & Mutisya, 2013), *Eucalyptus tereticornis*, *Tagetes minuta,* and *Carica papaya* (Muzemu, Chitamba, & Mutetwa, 2013); cheese wood, lemon‐scented gum, ginger, lime, mint, and tobacco (Longe, 2016); and ethanol extract of *Azadirachta indica* (*A. indica*), *Chenopodium ambrosioides*, *Melaleuca lanceolata*, and diatomaceous earth (Dekeba, Yetenayet, & Waktole, 2016), and many other researchers reported *A. indica* indicated a promising result as grain protectant against maize weevils (Issa, Afun, Mochiah, Owusuakyaw, & Braimah, 2011; Kifle, Mulatu, & Muluken, 2016; Shiberu & Negeri, 2017).

*Azadirachta indica* is rich in alkaloids (*azadiracthtin*) and other molecules such as salanine and melandriol after ingestion causes digestive disorders and loss of appetite (antifeedant activity) (Schmutterer, 1990). Neem seeds typically contain 0.2%–0.6% *azadirachtin* by weight, which need solvent extraction or other chemical processes to concentrate this active ingredient to the level of 10%–50% seen in the technical grade material used to produce commercial products (Isman, 2006). *Azadirachtin* is said to be highest in the kernel than in the leaves and other tissues of the plants (Rajapakse & Ratnasekera, 2010). It possesses antifeedant, repellant, growth disrupting, and larvicidal properties against a large number of pests (Nukenine, Chouka, Vabi, Reichmuth, & Adler, 2013).

#### 2.1.3 The use of underground pit storage

Producers in most parts of the country, especially around Oromia and southern nation and nationality people regions, use underground pit storage (Sertse & Disasa, 2014). This pit is dug in a flask shape with its top part narrower than bottom. The cereal grains can be stored for several years in a pit. The pit must stay sealed until the grain is needed for consumption or market. The risk of insect pests in underground pit storage is minor; however, the grains stored in these structures are usually heavily infected with mold. The cereals could be stored either as cluster or as grains in underground storage structures. Clusters are well compact in the store, and the store is sealed to reduce the possibility of air entry. Nowadays, few farmers have started to use big plastic sheets to plaster the entire surface of the underground storage to control moisture seepage and reduce mold infestation (Sertse & Disasa, 2014).

### 2.2 Preparation of indigenous food of Ethiopia

#### 2.2.1 Milk and milk products

Milk is a fluid that obtained from female mammals after secreted in their mammary glands. Nutritionally, it constitutes nearly all the required nutrients to sustain the life of the offspring. Human being has been used since ancient time the milks of different animals such goats, sheep, and cows as a food. Today, the term “milk” is referred to the cow's milk, while the milk of other animals is coined for instance as sheep milk or goat milk, when supplied commercially (Belitz, Grosch, & Schieberle, 2009). Thus, milk and milk products play a significant role in human nutrition around the globe (Bekele, Fekadu, & Mitiku, 2015). The demand of milk and milk products is increased in tropical areas including Ethiopia due to growth of people's income (Gemechu & Tola, 2017) and enhanced awareness for its nutritional benefit.

**Figure 2:**
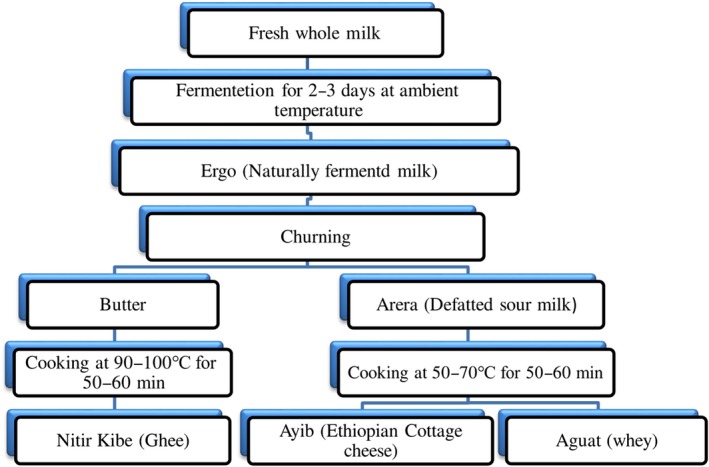
Flow diagram of fresh whole milk processing into indigenous milk‐based products in Ethiopia

Milk is among the highly perishable food product and can be also easily adulterated, while the quality of the milk is highly dependent on farm management (Bekele et al., 2015). Because of its perish‐ability milk is processed into different dairy products. In Ethiopia, dairy products are manufactured and consumed in many parts following the traditional processing step indicated in Figure 2. Fresh whole milk, whole sour milk (*Ergo*), butter, *Arera* (defatted sour milk), *Ayib* (a traditional cottage cheese), and *nitirkibe* (ghee) are the major dairy products produced and consumed in many parts of the country. The different reports indicated that depending on the type of milk product and preservation methods applied, traditionally processed dairy products can be stored for more than 2 years at ambient temperature. For instance, a study conducted by Seifu and Tassew (2014) in Bahr Dar reported that the storage life of sour milk (Ergo) varies between 3 and 5.2 days, Ayib between 2.1 and 3.7 days, unspiced butter between 8.6 and 18.8 months, and spiced butter between 4.5 and 18.3 months, whereas the storage life of ghee varies between 9.4 and 29 months at an ambient temperature. Another study conducted in Eastern Wollega reported that the shelf life of butter was extended up to 3.7 months at ambient temperature by the simple addition of salts (Gemechu & Tola, 2017).

Many of these products are produced using artisanal technologies on‐farm, and the types and processing steps of these dairy products can vary considerably from one area to the other (Seifu & Tassew, 2014). Like other countries, Ethiopians have been using milk products such as butter and ghee as part of their diet since prehistoric times. Despite milk's contribution to gross domestic product and value of butter as a food, sub‐Saharan Africa in general and Ethiopia, in particular, has failed to attain sufficiency in dairy products (Gemechu & Tola, 2017).

##### Whole sour milk (Ergo)

**Figure 3:**
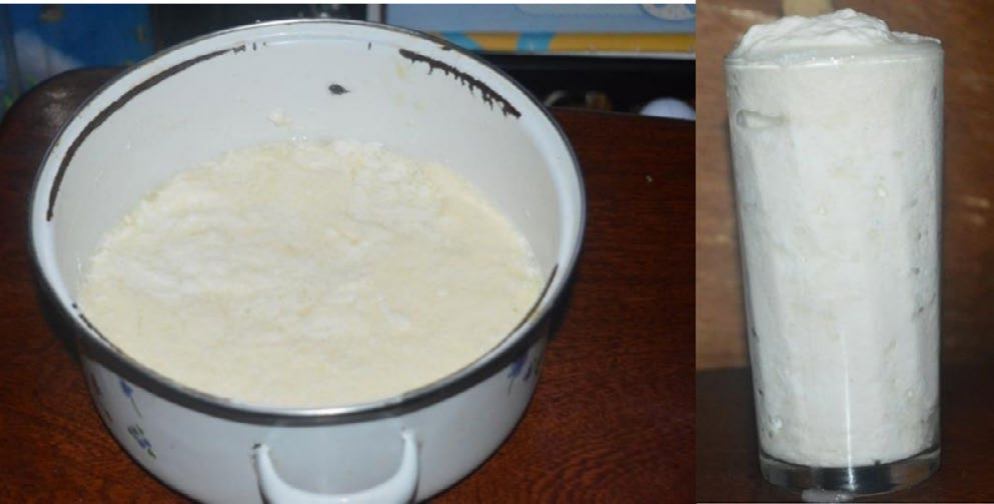
Ethiopian whole sour milk (Ergo)

It is common to prepare Ergo (Figure 3) in every household who are raring cattle's in both rural and semi‐urban area of Ethiopia. However, in the cities of Ethiopia including the capital city Ergo can be obtained in some cafeterias, small restaurants, and shops. Ergo is a naturally fermented whole milk products (Gemechu & Tola, 2017). The preparation practices of Ergo may slightly vary from place to place according to their cultural value and available resources, while in all places the product is produced based on the principle of natural fermentation. Specifically, in majority of rural area people clean the milking vessels and fermentation containers with locally available leaves such as bsana leaf (*Croton macrostachyus*) and nacha leaf (*Hibiscus macranthus Hochst. ex A. Rich)* (Chekole, Asfaw, & Kelbessa, 2015) using warm water. Then, they smoke the container with smoking plants such as cheba (*Acacia* spp.)*,* Woyira (*Olea Africana*), Dokima, Embuay (*Solanum incanum*), or maize cob to facilitate the fermentation process and promote taste and aroma of the milk products (Bekele et al., 2015). After smoking is completed, they collect the milk in the container and allowed it to ferment for 2–3 days to make it ready for whole sour milk consumption or further processing.

##### Butter

**Figure 4:**
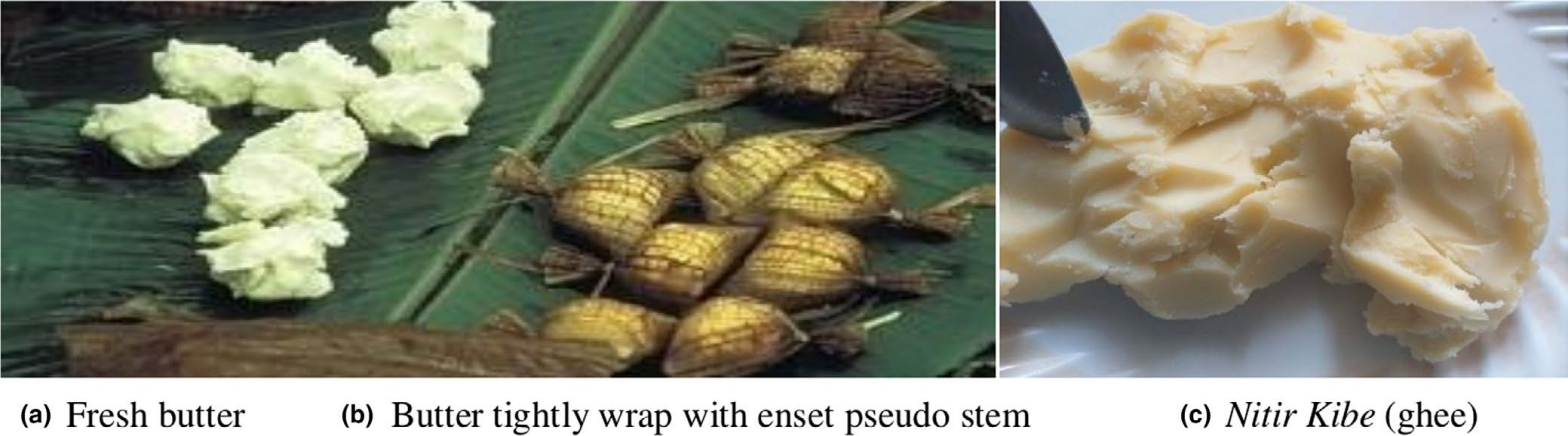
Fresh butter (a), butter tightly wrapped with Enset pseudostem (b), and Ethiopian *nitir kibe* (ghee) (c)

Butter is one of the prestigious products among the rural community. Besides consumption, people are using butter as traditional cosmetic products for keeping their beauty and moisturize their body and hair. Three types of butter are traditionally produced in Ethiopia, namely *Lega, Mekakelegna, and Besal* referring to fresh, semi‐rancid, and rancid butter, respectively, based on the degree of its lipolysis (Gemechu & Tola, 2017). Butter is traditionally produced by churning the whole sour milk, which was collected over several days in clay pot (churn) (Bekele et al., 2015; Bereda, Yilma, & Nurfeta, 2013). The churning and making of the butter is entirely done by women. Women detect the formation of the butter either by observing the change of sounds of the milk at the breakpoint (a point when the butter starts to form) or by inserting straw into the churn through the vent to see the adherence of tiny butter grains on its surface (Kefyalew, Solomon, Mitku, & Getachew, 2016). When the churning is done, the butter grains are collected and form lumps of butter, which are skimmed off. The butter is then kneaded in cold water and washed to remove visible residual buttermilk. The women used the butter are either for home consumption or sold to the market to fulfill the other needs of their family. To extend the shelf life of the butter, women store fresh butter (Figure 4a) in cool place after they tightly wrap it on the skin of the dried pseudostem of Enset (Figure 4b) or make *Nitir Kibe* (ghee) by melting and adding different spices (Figure 4c). The *Nitirkibe* is clarified and stored in cool place to solidify and further extend its shelf life.

##### Cottage type cheese (Ayib)

**Figure 5:**
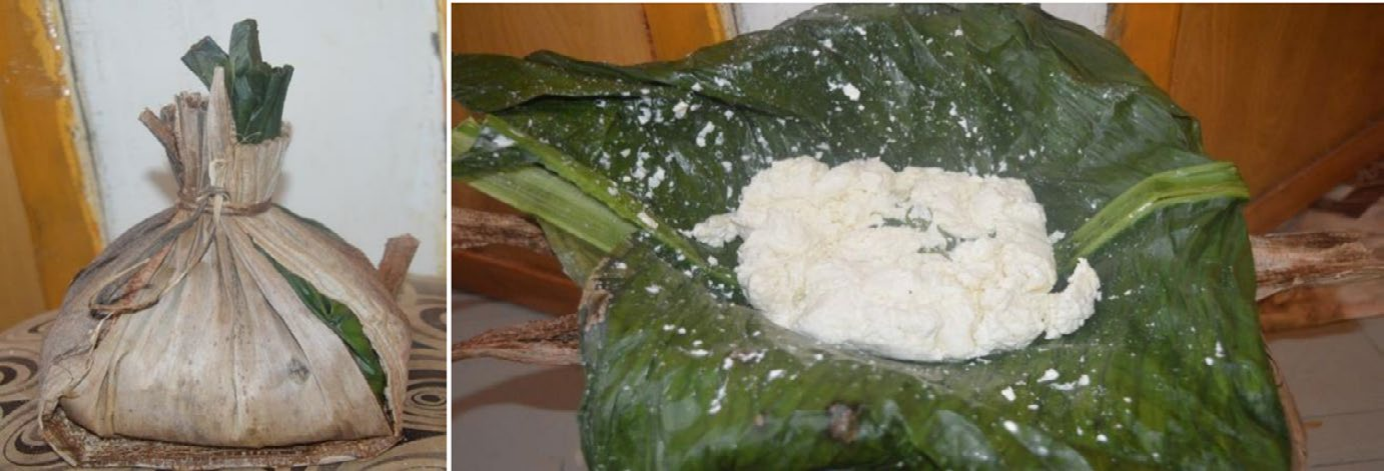
Ethiopian cottage cheese

To produce cottage cheese (Figure 5), buttermilk is slowly heated either by clay pot or stainless pan until it forms a curd (Bereda et al., 2013). Buttermilk is obtained after removal of the butter after churning of the whole sour milk. Locally, it is called *Arera*, which is either directly consumed within the family or heated to get cottage cheeses and its’ by‐product whey, which is locally known as *aguat* (Negash, 2018). Once the required level of curd formation is achieved, it is left for some time to cool down and the cheese is separated from the whey (Bekele et al., 2015). The whey (*aguat*) used for consumption by the majority of poor society living in rural area, while in semi‐urban area it is discarded. To extend the shelf life of the Ayib in addition to small amount of salt, women used to add different spices mainly Koserete (*Ocimum hardiense*), Tikure azmude (*Nigella sativa*), Korerima (*Aframomum angustifolium*), Tena Adam (*Ruta chalepensis*), and Abish (*Trigonela fenum*) (Bereda et al., 2013).

#### 2.2.2 Enset‐based products

Many wild species of Enset are distributed throughout central, eastern, and southern Africa as well as Asia. Records suggest that Enset has been grown in Ethiopia for more than 10,000 years (Jacob, 2004). Bezuneh (2010) noted that Enset has been cultivated as a food and fiber crop in Ethiopia for several years, and over 80% of the production is concentrated in the south and southwestern part of the country. It is ranked as the first in its importance as cultivated staple food crop in the highlands of central, south, and southwestern Ethiopia (Karssa, Ali, & Gobena, 2014; Tsegaye, 2002). Different researchers suggested that the crop is recognized as a food security crop and as traditional medicine (Bekele & Reddy, 2015; Brandt et al., 1997; Funte, Negesse, & Legesse, 2009; Nyunja, Onyango, & Erwin, 2009; Tsegaye & Struik, 2001; Tsehaye & Kebebew, 2006).

Enset is a crop that tolerates harsh environmental conditions including prolonged drought periods, flooding, and many diseases. Due to its drought tolerance, it is regarded as a priority crop in Ethiopia, where it makes a major contribution to the food security of the country. According to the 2014 agricultural sample survey report by the Central Statistics Agency of Ethiopia, it is estimated that about 35% of Ethiopians people use Enset products as staple foods (Country STAT Ethiopia, 2016).

Kocho (obtained by squeezing the pseudostem and fermenting it) and corm (the underground stem) are the main food source from Enset. The corm (the underground stem) can be cooked like an enormous potato, weighing up to 70 kg (Brandt et al., 1997). Those edible parts of Enset yield a highly carbohydrate‐rich staple or costaple food for 35% of Ethiopians people that inhabits the south and southwestern part of the country. Furthermore, a study on antioxidant capacity, total phenolics, and nutritional content in selected Ethiopian staple food ingredients revealed that Enset and its products have a potential for developing value‐added food products, which have nutritional and health benefit components (Forsido, Rupasinghe, & Astatkie, 2013).

**Figure 6:**
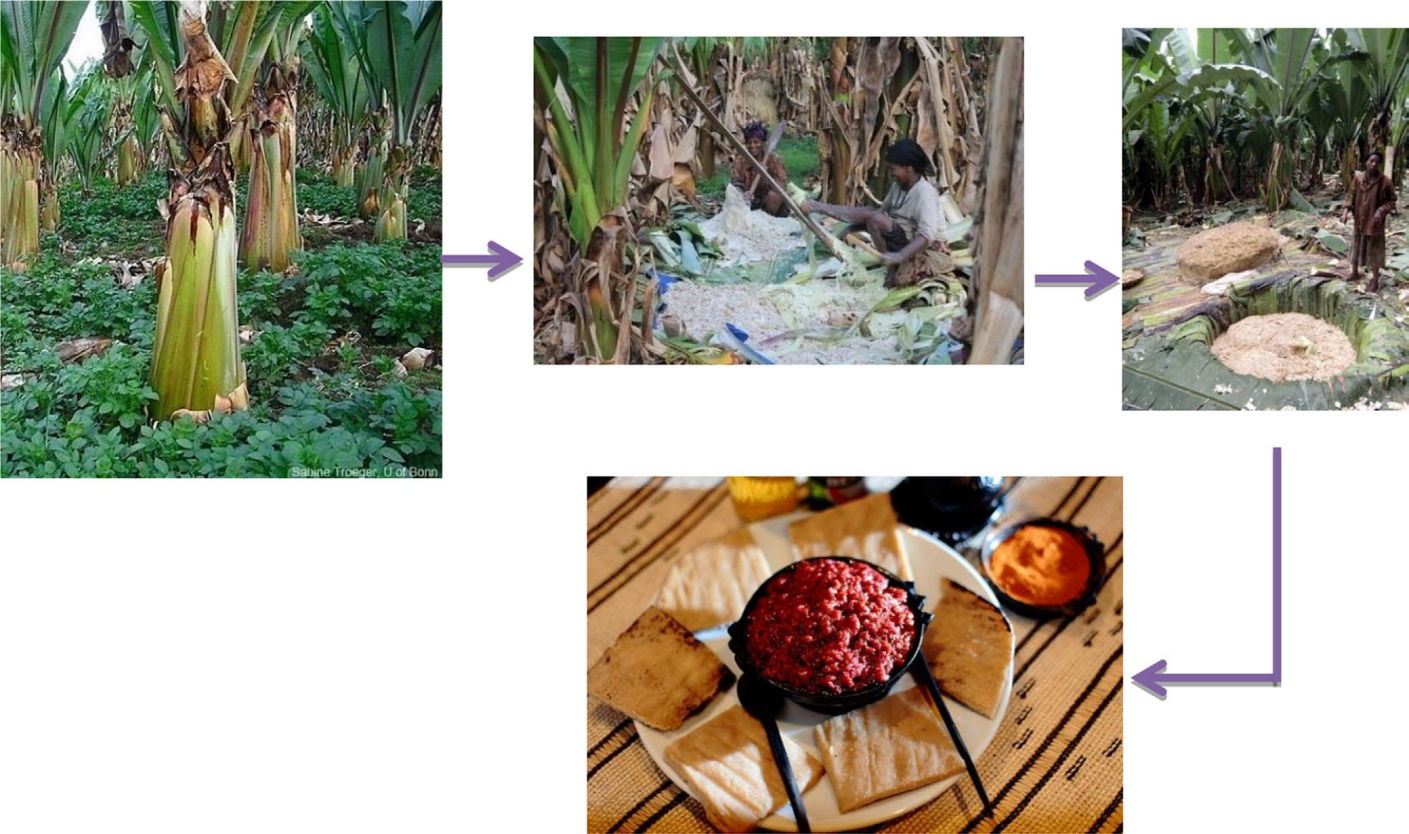
Enset‐based food product preparation flow diagram

The use and adaption of indigenous technical knowledge is invaluable in processing and preparation of Enset‐based food products. Enset‐based food product preparation is very time‐intensive and has several socio‐economic burdens, mostly on women who are usually responsible to prepare it. Its preparation involves cutting and harvesting the mature plant, digging, and lining a pit for fermenting the pulverized corm or root, scrapping (decorticating) the outer sheaths of the stem to remove edible parts, fermenting for 2–6 months as indicated in Figure 6. Finally, it can then be baked and eaten (Hunduma & Ashenafi, 2011). Foods prepared from Enset include bread, ferfer (a type of flatbread or injera), and porridge, which may contain meat and/or vegetables. It is even possible to prepare alcoholic or nonalcoholic drinks.

Even though it has been demonstrated the potential to integrate Enset into the marginal, drought‐prone farming systems of the country where malnutrition is prevalent still it remains an underutilized commodity in other parts, especially in center and northern part of the country because of limited information on its wider food uses.

#### 2.2.3 Cheka

*Cheka* is a cereal and vegetable‐based fermented beverage, which is consumed in southwestern parts of Ethiopia mainly in Dirashe and Konso. In these areas, people of all ages including infants, pregnant, and lactating women drink *cheka*. Worku, Woldegiorgis, and Gemeda (2015) indicated that most people particularly adults’ start drinking it early on an empty stomach and people in Konso on average drink 3–5 L of *cheka* per day but in Dirashe adults can drink up to 8 L per day.

Konso is the primary origin of *cheka* from which it is distributed to the neighboring areas around it, due to its suitability to perform high energy‐requiring activities like farming. Derashe people are the second next to Konso people to use *cheka*. As the majority of people in southern area are farmers, their work needs high energy, and once they consumed *cheka*, they do not fill any tiredness and not easily affected by disease, being fun and sociable. This makes difficult for them to stay without *cheka*. It is their special food which they want to have it every time and everywhere (Hailemariam, 2017).

**Figure 7:**
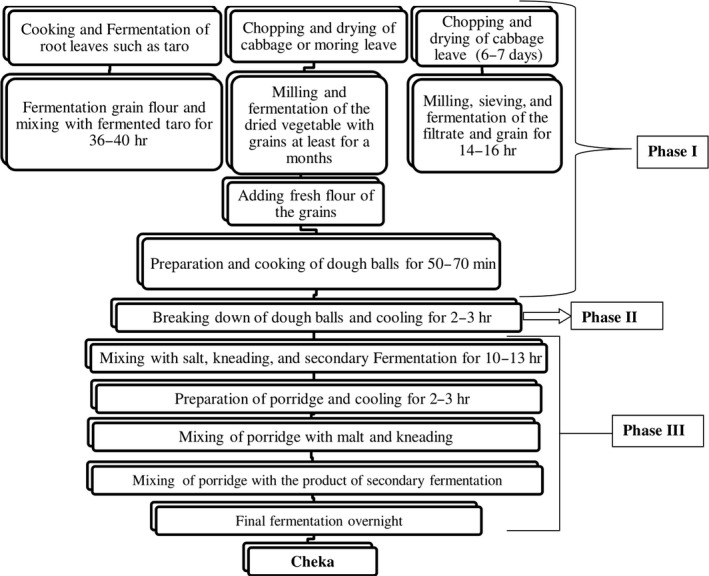
Flowchart for *cheka* preparation in most part of southern nations

**Figure 8:**
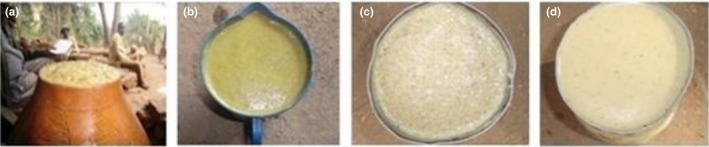
*Cheka* prepared from different types of vegetables and wild edible plants (Worku *et al.,* 2015)

Today, *cheka* is found almost in all parts of southern Ethiopia due to its dense energy, which support to do hard works, easily availability of its ingredients and low cost. The ingredients and methods of preparation of *cheka* slightly differ from place to place (Hailemariam, 2017; Worku et al., 2015). It is mainly prepared from cereals such as sorghum and maize (48.9%) and vegetables such as leaf cabbage, and moringa. In some areas, root and leaf of taro and edible leftover of Injera (Ethiopian flatbread) and Kitta are used rarely as ingredients to prepare *cheka*. Sometimes wild leafy edible plants could substitute cabbage and taro to prepare *cheka* drinks. The flow diagram to prepare *cheka* in most part of the southern nation region is indicated in Figure 7, while Figure 8(a‐d) shows readymade *cheka* from different ingredients at different districts of southern nation region.

#### 2.2.4 Injera

**Figure 9:**
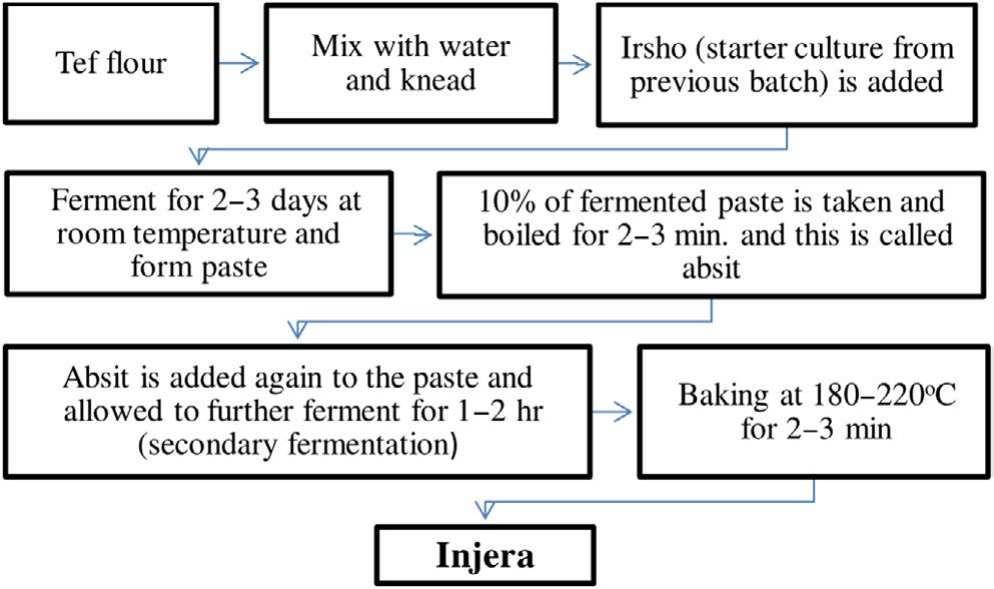
Injera preparation flow diagrams

Injera (Ethiopian flatbread) is commonly made in most of Ethiopian kitchen through different processing steps as per traditional practices inherited from parents. To prepare Injera (Figure 9), tef (*Eragrostis tef*) flour is mixed with water to make dough and then triggering a fermentation process by inoculating the dough with *ersho* (a starter culture, leftover from a previous fermentation). The starter culture is usually added at a ratio of 1:1.6w/v (Baye, Mouquet‐Rivier, Icard‐Vernière, Rochette, & Guyot, 2013; Yetneberk, Kock, Rooney, & Taylor, 2004).

**Figure 10:**
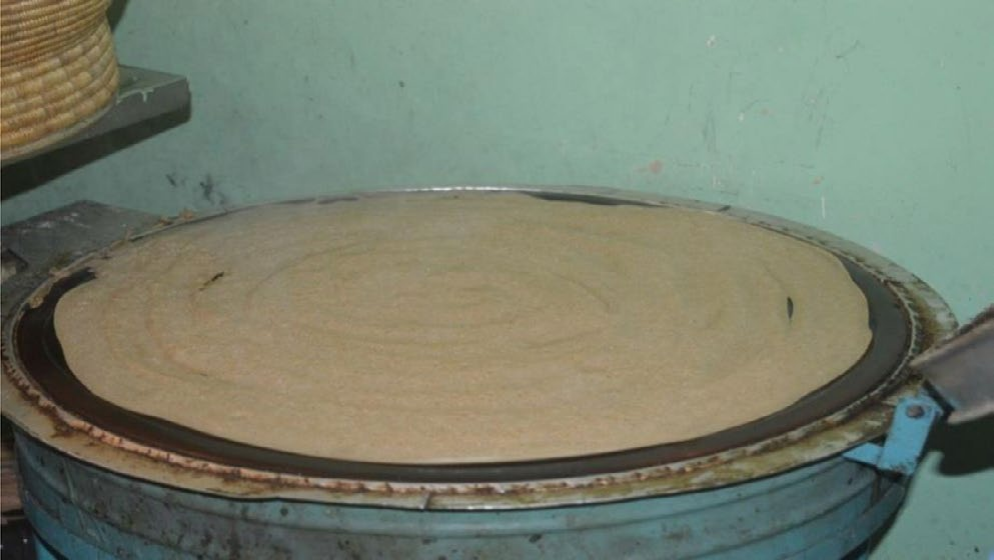
Injera baking process on *Eelee* or *Mixad* (open platter)

In Ethiopia, Injera‐making process consists of two stages of natural fermentation, which lasts within 2–3 days depending on the weather conditions (Dijkstra, Polman, Wulfften‐Palthe, Gamboa, & Ekris, 2008). The first step of the fermentation process in Injera making starts after the addition ersho to the dough. The initial 18 hr of the fermentation process are characterized by vigorous evolution of gas and maximum dough rising (Gashe, 1985). This is followed by the appearance of an acidic yellowish liquid on the surface of the dough at about 30–33 hr of the fermentation process (Ashenafi, 2006). Gas evolution decreases after the pH has fallen below 5.8 within 31 hr the fermentation process (Gashe, 1985). The liquid layer is discarded at the end of the first stage of fermentation. As soon as the liquid layer is poured off, about 10% of the fermenting dough is mixed with three parts of water and boiled for 5 min (Ashenafi, 2006). This is called absit (a dough enhancer) and mixed with the rest of the dough in the fermentation vat (Sahlin & Nair, 2012). Maximum dough rising, which normally takes 30 min to 2 hr, signals the termination of the second stage of the fermentation process (Dijkstra et al., 2008). At this stage, the dough is thinned with water into a batter before baking on an open platter. Finally, Injera (Ethiopian flatbread) is baked on open platter, which is called Eelee or Mixad in Ethiopian language as indicated in Figure 10. Baking is performed for 2–3 min at about 200–250°C.

As staple food, in every home of Ethiopians’ particularly in urban community, Injera is baked once in every 3‐day intervals. This is mainly due to their high perishable natures, which gets spoiled and lose its quality very fast. Its perish ability is mainly associated with its high moisture and nutrient contents, which supports the early growth and spoilage of mold. Mold growth and lose in texture are the two main factors, which makes Injera unsafe and less acceptable for human consumption approximately after 3–4 days of ambient storage. These conditions commonly happened due to its improper storage materials (traditional basket container called *messob*) that enhance the growth of mold and migration of moisture.

However, Ashagrie and Abate (2013) in his study explored ways to help preserve it longer. He used four chemical preservatives at the concentration of 0.3% calcium propionate, 0.2% potassium sorbate, 0.1% benzoic acid, 0.1% sodium benzoate, and 0.2% blended Injera batters just immediately before baking, and the preservatives used had prolonged the shelf life of Injera for up to 12 days. This will allow a continuous supply of safe and good quality Injera for both local and export markets. The postprocessing loss will be reduced and help producers to produce and sell more Injera. Consumers also will benefit from extended storage time, which enables them to save their time, energy, and labor costs for making or purchasing of Injera.

#### 2.2.5 *Buna qala*a (slaughtering coffee)

Coffee may be a crucial trade goods used around the world in numerous ways and has been an essentially vital for social, economic, political, and ritual physical objects for many centuries. Although there is presently nothing to substantiate such beliefs, it is acknowledged that near to the ground is endemic to Ethiopia and it is presupposed that the Oromo were the primary people to recognize its stimulating effect (Haberland, 1963; Jacobson‐Widding & Beek, 1990; Wild, 2005; Yedes, Clamons, & Osman, 2004).

The eating of coffee among the Oromo people goes back to the time of immemorial when it was eaten for its energizing effect (Weinberg & Bealer, 2004). According to verbal traditions, the Oromo people ate food processed from coffee beans. They collected the ripe near to the ground berries, from coffee trees and ground them with stone mortars, and mixed the mashed seeds and pulp with butter from which they formed small balls that they carried for subsistence during long journeys. The Oromo use of coffee as a meal continues to this date (Bartels, 1983; Baxter et al., 1996; Weinberg & Bealer, 2004; Wild, 2005). *Buna qalaa* is the common coffee meal that has continued to the present time. Wild (Sahlin & Nair, 2012) also reported that the *buna qalaa* were eaten by warriors, farmers, and merchants faced with hard work or long journeys by which they were able to overcome the problems of hunger and exhaustion.

**Figure 11:**
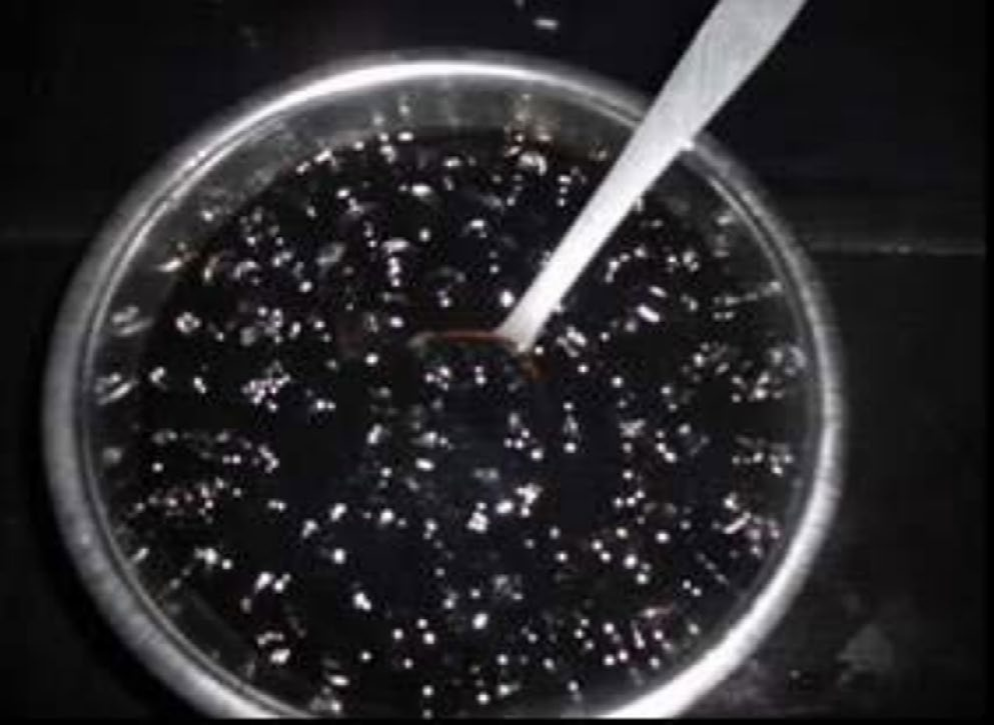
*Buna qalaa* ready for eating

Buna qalaa (Figure 11) is prepared of green or dried coffee berries, which are washed and opened by the teeth. Opened low berries square measure adscititious and cooked during a pot ablaze on hearthstones. Pure butter is added to the toasted coffee beans while still on fire. Thus, *buna qalaa* is not simply a particular marker of Oromo people's food habit, but a unique achievement of the society in the sphere of utilizing coffee for dietetic use. The Oromo people in their long‐distance travel or on any campaigns traverse in immense deserts, carrying nothing to eat with them but the berries of the coffee tree roasted, pulverized and then combined with butter to an exact consistency that allow its being rolled into lots and packed and carried in leathern gags until required for use. They claim that one of these balls can support them for a full day, throughout their travels or in active war, higher than a loaf of bread or a meal of meat, because it cheers their spirits besides satisfying their appetite (Weinberg & Bealer, 2004). Once readymade, *buna qalaa* can stay for more than 2 years without significant quality loss under ambient conditions (Baxter et al., 1996; Weinberg & Bealer, 2004).

#### 2.2.6 Wild edible plant‐based foods

Ethiopian residents, especially the rural people, are endowed with a deep knowledge concerning the use of wild plants. Consumption of wild edible plants is an important local survival strategy and made necessary by climatic fluctuations that hamper agricultural efforts (Gemedo‐Dalle, Maass, & Isselstein, 2005). Increased consumption of wild edible plants enables people to cope better with erratic weather, untimely rains, and seasonal droughts (Feyssa, Njoka, Asfaw, & Nyangito, 2011; Gemedo‐Dalle et al., 2005). Guinand and Lemessa (2001), and Balemie and Kebebew (2006) reported that people in Southern Ethiopia, Konso, Derashe, and Burji districts still use a diversity of wild edible plants. In this area, most of the edible fruits and seeds are collected and immediately used by children. These wild edible plants provide essential supplies of vitamins and minerals particularly to the children (Asfaw & Tadesse, 2001). Dias and Ryder (2011) also reported wild edible plant plays a significant role in human nutrition, as sources of phytonutrients such as vitamins, minerals, dietary fiber, and bioactive phenolic compounds. Furthermore, wild edible plants have been strongly associated with overall good health, improvement of gastrointestinal health and vision, reduced risk for some forms of cancer, cardiovascular diseases, heart diseases, stroke, diabetes, anemia, gastric ulcer, rheumatoid arthritis, and other chronic diseases in humans (Keatinge et al., 2010; Mullie & Clarys, 2011).

Despite their importance as a food security crop and as sources of phytonutrients, people use these crops only when there is a serious shortage of food, which may affect all strata of the population from the poorest to the richest. During this period, dishes prepared from wild edible plants are consumed by all groups of the population. Leafy edible plants such as *Corchorus olitorius, Amaranthus caudatus*, *Portulaca quadrifida, Moringa stenopetala,* *Leptadenia hastata*, *Solanum macrocarpon*, *Ficus* *sycomorus*, *Sclerocarya birrea*, *Syzygium guineense,* and *Ximenia americana* are collected by women and young girls and used like cabbage, fresh fruit or fruit juice, hot drink, boiled or roasted grain, and tuber (Balemie & Kebebew, 2006). Some wild species are used as coffee and tea substitutes or used as roasted/boiled grain. For example, leaves of *Lanatana rhodesiensis* are roasted on iron plate, pounded, and then boiled in clay pot for use as hot tea to reduce the feeling of starvation. However, most of the leafy edibles are consumed after being prepared as *kurkufa*. In preparing *kurkufa*, small boluses, which are made from unleavened dough of maize, sorghum, or barely, are added into clay pot/jar containing boiled leafy vegetables and leftover on fire. When *kurkufa* matures, excess water is drained off, salted, and mixed with butter to improve the flavor. The *kurkufa* is then put on to *toma*, local bowl made usually from *Ficus *spp. and served along with unleavened bread (*kita*) or leavened bread (Balemie & Kebebew, 2006).

### 2.3 Contribution of Ethiopian indigenous food in attainment of food security

It is recognized that indigenous foods and dietary diversity within an ecosystem can be powerful sources of nutrients and benefits human health (Ghosh‐Jerath, Singh, Magsumbol, Kamboj, & Goldberg, 2016). Despite this fact, the use of indigenous foods has declined due to the negligence of the potential of these foods in modern commercialized and industrialized markets and lack of investment in research and development.

In Ethiopia, during peak harvest periods the prices of agricultural products drop to as low as 6–20 USD per 100 kg bag of products depending on product type, but can hike to as high as 36–50 USD during scarcity. The low prices have discouraged farmers from harvesting and selling their crop, thus causing heavy losses (Ray & Ravi, 2007). Besides, the country is not getting foreign exchange from agricultural products as expected due to limited application of postharvest technology, which makes the product of inferior quality, with less chance of competing in the world market. There are no enough processing plants, and the country is losing foreign currency by importing processed products from abroad.

Postharvest losses reduction and product commercialization could be possible by promoting indigenous food handling, processing, and preservation techniques with the view of upgrading them. Ethiopian indigenous food handling, processing, and preservation techniques are simple to operate and low cost and could be a basis for small‐scale food‐processing industries. Moreover, there are underutilized indigenous crops in the country that can be commercially produced and compliment food and nutrition security efforts of the country. Commercialization and use of underutilized indigenous food sources enhance food supply, promote diversity of diets, and would serve as a source of income for producers. However, the knowledge of exploiting underutilized food is often undervalued that has consequently led to the loss of some of them. Therefore, creating a value chain with appropriate technologies and adding alternative value to the edible parts of underutilized food is an important issue in addressing food security.

The consumption of Ethiopian indigenous food contributes to the prevention of many diseases and conditions that can result from an unbalanced diet. The consumption of Injera (Ethiopian flatbread) as a staple food contributes to the prevention of many diseases like anemia, obesity, bone disease, and diabetes. According to Bultosa and Taylor (2004), the proximate composition (db) of Tef is reported to be 9.4%–13.3% protein, 73% carbohydrate, 1.98%–3.5% crude fiber, 2.0%–3.1% fat, and 2.7%–3.0% ash. It is rich in calcium, iron, and protein, and possesses an impressive set of amino acids. Furthermore, its sodium, saturated fat, and cholesterol contents are low as compared to other cereals. Additionally, it does not contain gluten and is an increasingly important dietary component for individuals who suffer from gluten intolerance (Boka, Woldegiorgis, & Haki, 2013). Furthermore, a study on antioxidant capacity, total phenolics and nutritional content in selected Ethiopian staple food ingredients revealed that Tef has a potential for developing value‐added food products with nutritional and health benefit components (Forsido et al., 2013).

Other indigenous Ethiopian foods such as *buna qalaa‐, cheka‐,* and *enset‐*based products are consumed by farmers to do hard works, and once they consume this food, they will not fill any tiredness, and not easily affected by disease, being fun and sociable. Mohammed, Gabel, and Karlsson (2013) reported that dry matter content of Enset is 11%–15% of fresh weight and the organic matter fraction of dry matter is around 90%. As fractions of dry matter, crude protein content of 3%–13%, crude fat 0.4%–5%, crude fiber 6%–24%, and soluble carbohydrates 50%–80% is recorded for different parts of Enset. Although they are nutritionally important and an important food security crops, their investigation has not received the scientific attention that they deserve in the country. Studying their nutritional value, microbial dynamics as well as understanding process variables and properties of raw materials during their preparations will help in making them as a commercially viable as possible.

## 3 CONCLUSION

To improve food security, safe and economic use of the available food, and avoiding wastage or loss is very important. Indigenous knowledge of food processing, preservation, and storage plays a vital role to help all stakeholders to maintain product safety and support the country in the reduction of food insecurity problems. As poor rural households do not have the necessary facilities and technology to process, preserve, and store food, they have to use their indigenous knowledge that has been passed on from generation to generation. These indigenous knowledge and practices are simple to operate and accessible and could improve the food insecurity problem if they could be upgraded to faster and better practices. Therefore, upgrading indigenous knowledge of food processing, preservation, and storage through incorporating into any policy of program geared toward reduction of food insecurity will boost the people confidence and their ability to be part of the solutions to the challenges facing them.

## CONFLICT OF INTEREST

All authors declare no conflict of interest.

## AUTHORS’ CONTRIBUTIONS

The first author was responsible for the accomplishment of most of the works, searching literature data and write up of the paper. The second author also contributed in the manuscript preparation and standardized the paper. Both authors contributed equally to the preparation of the manuscript and approved the final manuscript for publication.

## ETHICAL STATEMENT

This study does not involve any human or animal testings.
